# Systemic inflammatory profiles are associated with long-term kidney failure and patient mortality in chronic kidney disease

**DOI:** 10.1093/ckj/sfaf352

**Published:** 2025-12-09

**Authors:** Alberto Martínez-Castelao, Takehiro Hasegawa, Beatriz Fernández-Fernández, José Luis Górriz, Alberto Ortiz, Maria Quero, Gema Fernández-Fresnedo, Jorge-Iván Zamora, Maria Jose Soler, José-Carlos Primo-Alvarez, Nicolas Roberto Robles, Norma Inés Venegas, Felipe Ignacio Ojeda, Antonio Gil-Paraíso, Irene Maria Llorente-Cortijo, Caridad Martínez-Villanueva, Esteban Poch, Jinny Sanchez-Rodriguez, Yun Li, Saruta Yuko, Juan F Navarro-González

**Affiliations:** Nefrología, Hospital Universitari de Bellvitge, Hospitalet, Barcelona, Spain; RICORS2040, Madrid, Spain; Sysmex R&D Center Europe GmbH, Hamburg, Germany; RICORS2040, Madrid, Spain; Department of Nephrology and Hypertension, Health Research Institute-Fundación Jiménez Díaz University Hospital, Universidad Autónoma de Madrid (IIS-FJD, UAM), Madrid, Spain; Departamento de Medicina, Facultad de Medicina, Universidad Autónoma de Madrid, Madrid, Spain; RICORS2040, Madrid, Spain; Servicio de Nefrología, Hospital Clínico Universitario Valencia, INCLIVA, Universitat de València, Valencia, Spain; GEENDIAB; RICORS2040, Madrid, Spain; Department of Nephrology and Hypertension, Health Research Institute-Fundación Jiménez Díaz University Hospital, Universidad Autónoma de Madrid (IIS-FJD, UAM), Madrid, Spain; Departamento de Medicina, Facultad de Medicina, Universidad Autónoma de Madrid, Madrid, Spain; Nefrología, Hospital Universitari de Bellvitge, Hospitalet, Barcelona, Spain; Servicio de Nefrología, Hospital Universitario Marqués de Valdecilla, Cantabria, Spain; Servicio de Nefrología, Hospital Universitari Vall Hebrón, Barcelona, Spain; RICORS2040, Madrid, Spain; GEENDIAB; Servicio de Nefrología, Hospital Universitari Vall Hebrón, Barcelona, Spain; Servicio de Nefrología, Hospital Burela, Lugo, Spain; RICORS2040, Madrid, Spain; Servicio de Nefrología, Hospital Universitario Infanta Cristina, Badajoz, Spain; Servicio de Nefrología, Hospital Universitario Virgen de Arrixaca, Murcia, Spain; Hospital Universitario Son Espases, Palma de Mallorca, Spain; Servicio de Nefrología, Hospital Universitario San Pedro, Logroño, La Rioja, Spain; Servicio de Nefrología, Hospital Arquitecto Marcide, El Ferrol, Spain; Servicio de Nefrología, Hospital General de Valencia, Valencia, Spain; Servicio de Nefrología, Hospital Clínic, Barcelona, Spain; RICORS2040, Madrid, Spain; Department of Nephrology and Hypertension, Health Research Institute-Fundación Jiménez Díaz University Hospital, Universidad Autónoma de Madrid (IIS-FJD, UAM), Madrid, Spain; Sysmex R&D Center Europe GmbH, Hamburg, Germany; Sysmex R&D Center Europe GmbH, Hamburg, Germany; RICORS2040, Madrid, Spain; GEENDIAB; Servicio de Nefrología y Unidad de Investigación, Hospital Universitario Nuestra Señora de Candelaria, Tenerife, Spain

**Keywords:** chronic kidney disease, cytokines, diabetes mellitus, IL-22, outcomes, progression, systemic inflammation

## Abstract

**Background:**

Chronic kidney disease (CKD) is frequently associated with systemic inflammation. However, the relationship between inflammatory cytokines and CKD progression remains incompletely understood.

**Methods:**

The PROGRESER study (Factores de PROGRESion en Enfermedad Renal) was a multicentre, prospective observational cohort that included patients with CKD stage G3. A total of 17 plasma and 10 urinary inflammatory cytokines were measured in 165 participants, at baseline, 18 months and 36 months, using a fully automated HISCL immune analyser. Unsupervised cluster analysis identified six distinct patient clusters. Associations between cytokine levels and kidney outcomes [estimated glomerular filtration rate (eGFR), urinary albumin-to-creatinine ratio (uACR), KDIGO risk scores, kidney replacement therapy (KRT)], cardiovascular outcomes (cardiovascular hospitalization) and death was assessed during a follow-up of up to 10 years. The primary outcome was a combination of KRT or death.

**Results:**

Plasma levels of 15 cytokines and 6 urinary cytokines differed between patients with CKD and 30 healthy controls. Of these, plasma interleukin (IL)-8 levels were inversely associated with uACR slopes, while IL-22, tumour necrosis factor (TNF)-α and Growth Differentiation Factor 15 (GDF-15) levels showed positive correlations with uACR progression. Additionally, plasma TNF-α and GDF-15 were associated with KRT and/or death with 10 years.

Cluster analysis of plasma IL-8, IL-22, TNF-α and GDF-15 identified six distinct patient clusters. Clusters 3 (elevated levels of IL-22, TNF-α, GDF-15), 4 (elevated levels of all four cytokines) and the uncommon Cluster 5 (high GDF-15 only) were associated with worse kidney, cardiovascular and survival outcomes, while Cluster 6 (low cytokine levels) represented patients with slower disease progression and the best long-term outcomes. Over time, patients frequently transitioned to more severe clusters.

**Conclusion:**

In CKD G3 patients, systemic inflammation can be stratified using cytokine profiles. Specific cytokine clusters are linked to faster progression of CKD and cardiovascular disease, and to worse long-term outcomes including kidney failure and mortality.

KEY LEARNING POINTS **What was known:**

- Chronic kidney disease (CKD) progression is thought to be a combination of oxidative stress, systemic inflammation and tissue damage.
- No therapy targeting specific inflammatory cytokines is under clinical use in CKD outside immune-mediated diseases.
- Plasma and urine cytokines may provide information about outcomes and could be useful in development of new drugs.

**This study adds:**

- A development and evaluation of plasma and urine cytokines in patients with CKD stage G3 participating in a 3-year observational prospective Spanish study (the PROGRESER study) designed to investigate risk factors associated with CKD G3 progression.
- Evidence of systemic inflammation in patients with CKD G3 can be categorized into clusters that are associated with disease severity and hard outcomes.
- Provide a basis for identifying to characterize novel molecular mechanisms involved in CKD progression and in the systemic impact of CKD.

**Potential impact:**

- Our study provides further evidence supporting the critical role of systemic inflammation in CKD progression, demonstrating significant differences in cytokine levels between CKD patients and healthy controls, irrespective of diabetes status, and associated with outcomes.
- We have found a specific plasma cytokine cluster of four cytokines that may inform research into novel pathophysiological pathways as well as risk stratification and response to therapy.
- The six clusters identified in our manuscript may help design clinical trials of anti-inflammatory therapies targeting specific inflammatory cytokines.

## INTRODUCTION

Chronic kidney disease (CKD) is a major global public health issue [1]. The prevalence increases with age and population aging is magnifying the burden of CKD [2–5]. CKD may progress to kidney failure, requiring kidney replacement therapy (KRT), and is associated with increased risk of cardiovascular disease and premature mortality [6]. Indeed, CKD is projected to become the fifth global cause of death by 2040, and the third by 2050 in countries with long life expectancy such as Spain and Japan [7].

Despite advances in the understanding of pathophysiology and treatment of CKD, 180 000 patients still progress to kidney failure every year in Europe and the USA alone [2]. CKD G3 [estimated glomerular filtration rate (eGFR) 30–59 mL/min/1.73 m^2^] is the most common and controversial form of CKD [6]. It has been proposed that elderly (aged over 65 years) patients with eGFR between 45 and 60 mL/min/1.73 m^2^ should not be considered to have CKD in the absence of increased urinary albumin:creatinine ratio (uACR ≥30 mg/g, A1 albuminuria) [8]. Even for patients with eGFR <45 mL/min/1.73 m^2^, the mean rate of annual eGFR loss in the placebo arm of recent clinical trials was around –1 mL/min/1.73 m^2^ for those with A1 albuminuria and slightly faster (–1.7 mL/min/1.73 m^2^) for those with A2 albuminuria, meaning that many would never develop kidney failure [9]. Thus, further tools for risk stratification are needed in patients with CKD G3. Progression of CKD is thought to be driven by a combination of oxidative stress, systemic inflammation and tissue injury, as exemplified by diabetes mellitus (DM), the most common cause of CKD [10]. However, no therapy targeting specific inflammatory mediators is currently in clinical use for CKD of non-immune origin. Therefore, we hypothesized that assessing plasma and urinary cytokines may provide information on risk stratification based on pathogenic pathways related to inflammation that are not specifically targeted by current therapeutic approaches and may be useful for patient selection of future clinical trials.

To address this gap, the present study evaluated key inflammatory cytokines in plasma and urine in patients with CKD G3 with and without DM who participated in a 3-year prospective study in which cytokines were repeatedly assessed, and has now collected hard outcomes (kidney failure, mortality) at 10 years.

## MATERIALS AND METHODS

### Study population

The PROGRESER study (Factores de PROGRESion en Enfermedad Renal) is an observational, prospective, multicentre study designed to investigate risk factors associated with the progression of CKD G3. The study was conducted across 25 nephrology departments in Spain, with an initial follow-up period of 3 years [10]. It was approved by the Spanish Agency of Medicines and Medical Devices (AEMPS) on 29 September 2010 as a post-authorization observational study and by the Clinical Research Ethics Committee of the Hospital Universitari de Bellvitge, Hospitalet de Llobregat, Barcelona (study protocol PR 171/11), as well as by the ethics committees of all participating centres. Patients signed informed consents for enrollment in the study and for biobanking of biological samples. The primary objective was to assess the risk factors associated with CKD progression [3]. The secondary objectives included evaluation of hospitalization rates, mortality and related clinical factors.

### Inclusion and exclusion criteria

Inclusion criteria were age ≥18 years or older, CKD G3 defined as an eGFR between 59 and 30 mL/min/1.73 m^2^ in two successive determinations separated by at least 3 months using the Modification of Diet in Renal Disease equation, with A1–A3 albuminuria, a life expectancy >1 year and signed informed consent. Exclusion criteria were active malignancy, ongoing infection; proteinuria over 3 g/day; severe psychiatric illness or inability to sign informed consent; pregnancy or breastfeeding.

### Recruitment and follow-up

Patients were prospectively enrolled from nephrology outpatient clinics at participating centres between January 2012 and September 2015. The initial follow-up was 3 years. Clinical characteristics, eGFR and uACR, were assessed, and biobanked plasma and urine samples were collected at baseline. Thereafter, eGFR, uACR, and biobanked plasma and urine samples were collected at 18 and 36 months. At 10 years of follow-up, eGFR and uACR, patient survival, cause of death and need for KRT (dialysis or transplantation) were assessed.

### Outcome definition

For the present analysis, kidney and mortality outcomes were defined. The primary combined outcome was mortality at 10 years or kidney failure requiring KRT with either dialysis or kidney transplantation at 10 years (Supplementary data, Fig. S1). Secondary outcomes were the individual components of the primary outcome at 10 years. KRT or death, as well as death were evaluated for 157 participants (excluding those lost to follow-up). KRT was evaluated over 103 patients (excluding those lost to follow-up or dead).

Additional secondary outcomes were evidence of progression of CKD (uACR, eGFR) or cardiovascular disease (CVD) (hospitalization for CVD) at 36 months. Thus, uACR and eGFR slopes were calculated using at least three data points from baseline to 36 months and expressed in mg/g/year and mL/min/1.73 m^2^/year, respectively. Delta uACR and delta eGFR values were calculated as values at 36 months minus values at baseline. Kidney Disease: Improving Global Outcomes (KDIGO) risk category, based on uACR and eGFR values as shown in Supplementary data, Fig. S2 [6], was analysed for up to 36 months.

### Cytokine assessment

Seventeen plasma and 10 urinary cytokines biomarkers were measured using a fully automated immune analyser (HISCL-5000 or HISCL-800; Sysmex, Hyogo, Japan) [11]. Supplementary data, Table S1 presents assay characteristics. Interleukin (IL)-17 levels were measured using a fully automated highly sensitive immune analyser (HI-1000; Sysmex) [12]. Urine samples were processed using a sample extraction buffer (Sysmex, Hyogo, Japan) and then diluted with PBS containing 1% BSA or PBST, ensuring the appropriate dilution rate for each biomarker. In addition to PROGRESER participants, cytokine levels were measured in 30 healthy young individuals without any known medical condition from the IIS-FJD (Instituto de Investigación Sanitaria de la Fundación Jiménez Díaz) biobank. These data provide reference values for health.

### Statistical analysis

Statistical significance was set at *P*-values <.05 (two-tailed). The Fisher’s exact test, Steel–Dwass test and Kruskal–Wallis test were applied using R (version 4.0.3; R Foundation for Statistical Computing, Vienna, Austria). Associations between biomarker levels and gene expression pathways were analysed using Spearman’s rank based on baseline plasma and urine cytokine levels. Additionally, unsupervised hierarchical cluster analysis was performed using the City-block method and complete linkage with Cluster 3.0 (Human Genome Centre, University of Tokyo, Tokyo, Japan).

## RESULTS

### Characteristics of participants

PROGRESER enrolled 462 individuals with CKD G3, of whom 165 had biobanked plasma and urine samples and were enrolled in the present substudy (Table 1). Sixty-five participants had DM [60 type 2 DM (T2DM); 5 type 1 DM (T1DM)] and 100 did not. Median age was 68.0 years. Median eGFR was 42.8 mL/min/1.73 m² and uACR 42.7 mg/g.

**Table 1:** Baseline demographics and outcomes at 36 months (uACR, eGFR, hospital admissions) and 10 years (uACR, eGFR, deaths, KRT).

	CKD			Healthy			
	Median	Q1–Q3	*n*	Median	Q1–Q3	*n*	*P*-value
Age, years	68.0	61.0–74.0	165	30.5	28.0–38.0	28	<.0001
Gender, *n* (female/male)		126/39			2/26		<.0001
DM, *n* (%)		65 (39.4%)					
Hypertension, *N* (yes/no)		147/18					
Weight, kg	80.0	72.5–88.0	165				
SBP, mmHg	139.0	128.0–150.0	165				
DBP, mmHg	75.0	69.0–83.0	165				
MAP, mmHg	96.8	90.0–103.1	164				
eGFR and uACR: baseline and outcomes							
eGFR (MDRD, baseline), mL/min/1.73 m^2^	42.8	37.4–50.3	165	77.8	69.0–86.7	28	<.0001
eGFR (36 month), mL/min/1.73 m^2^	39.5	33.4–48.0	153				
eGFR (10 year), mL/min/1.73 m^2^	31.7	21.2–42.3	155				
Delta eGFR (MDRD) to 36 mo, mL/min/1.73 m^2^	–3.7	–10.9 to 1.8	165				
Slope eGFR (MDRD) to 36 months, mL/min/1.73 m^2^/year	–1.2	–3.1 to 0.6	165				
uACR (baseline), mg/g	42.7	10.4–284.4	123				
uACR (36 month), mg/g	113.5	32.5–446.5	140				
uACR (10 year), mg/g	111.5	35.0–666.8	138				
Delta uACR to 36 months, mg/g	8.3	–22.0 to 141.4	111				
Slope uACR to 36 months, mg/g/year	11.7	–1.8 to 93.2	152				
Other baseline biochemistry parameters							
Serum creatinine, mg/dL	1.6	1.4–1.8	165	0.9	0.8–1.0	28	<.0001
Urea, mg/dL	67.9	55.0–78.0	165				
Hemoglobin A1c, %	6.6	5.9–7.5	92				
Total cholesterol, mg/dL	179.9	151.0–205.0	165				
Triglycerides, mg/dL	121.8	89.0–191.0	152				
LDL cholesterol, mg/dL	107.0	85.3–128.0	110				
HDL cholesterol, mg/dL	46.5	36.0–57.1	114				
AST, mU/mL	22.0	18.0–28.0	125				
ALT, mU/mL	19.2	16.0–27.0	165				
Uric acid, mg/dL	7.0	5.8–8.1	165				
Ferritin, ng/mL	98.5	45.3–181.4	154				
Treatment for DM		32/33					
Insulin, *n* (yes/no)		48/17					
Oral antidiabetics, *n* (yes/no)							
Number of hospital admissions^a^, *n* (1/2/3/4/5/6/7/8)		29/11/3/4/3/0/0/2					
Hard outcome at 10 years							
Death or kidney replacement therapy (KRT), *n*/*N* (%)		75/157 (47.8%)					
Death, *n*/*N* (%)		54/157 (34.4%)					
KRT, *n*/*N* (%)^b^		21/103 (20.4%)					

Baseline characteristics of participants. The data are presented as median values with interquartile ranges (IQR), *N* for continuous variables and counts with percentages for categorical variables. The *P*-values were calculated using the Mann–Whitney U-Test or Fisher’s test.

^a^Hospital admissions recorded for 36 months.

^b^KRT as an isolated outcome could only be assessed in patients who were alive at 10 years.

KRT, dialysis or kidney transplantation.

MDRD, Modification of Diet in Renal Disease Studyl LDL, low-density lipoprotein; HDL, high-density lipoprotein; AST, aspartate aminotransferase; ALT, alanine aminotransferase; NA, not applicable.

### Patients with CKD G3 have evidence of systemic inflammation

**Figure 1:**
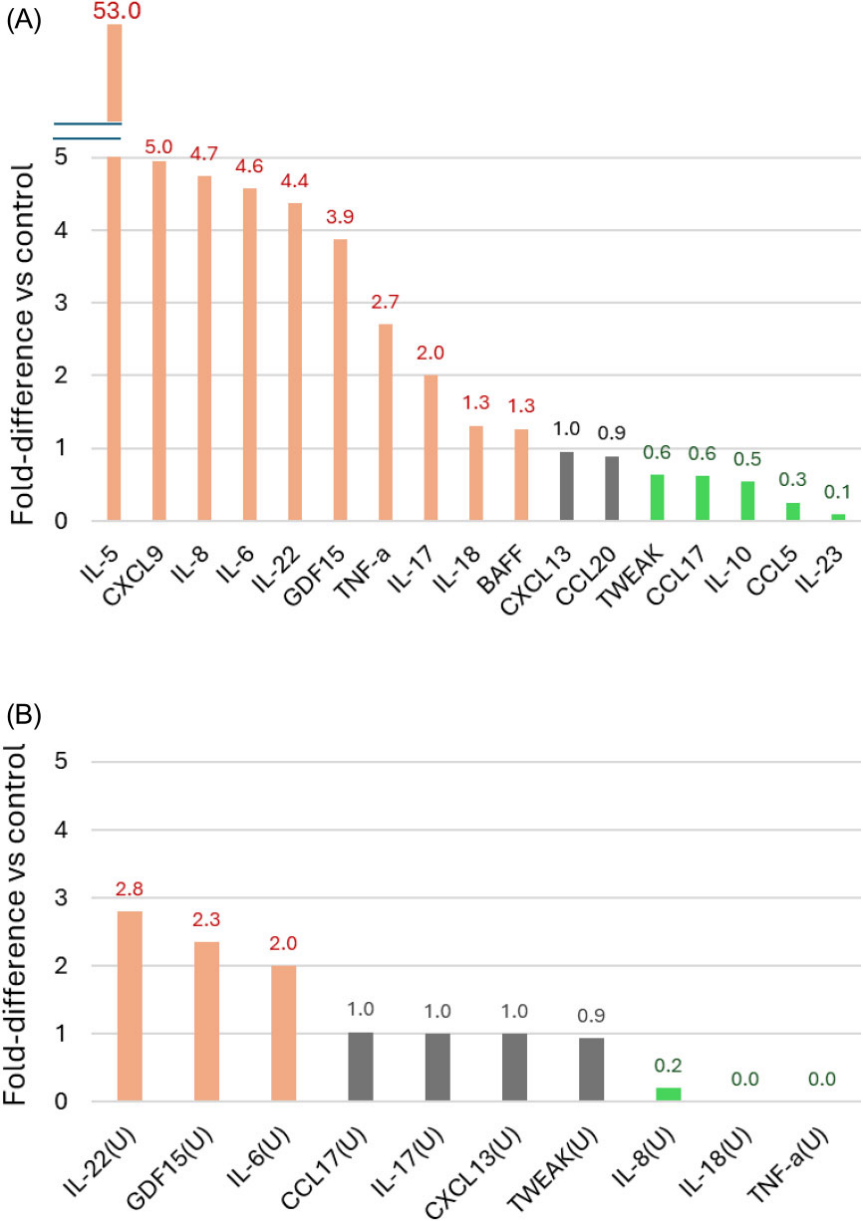
Cytokine levels in patients with CKD G3 versus healthy controls. Figure shows the fold-difference between median values in patients with CKD G3 and the median values in healthy controls. Detailed data on median and interquartile range as well as *P*-values are shown in Table 2 and Supplementary data, Fig. S3. (**A**) Plasma cytokines. (**B**) Urine cytokines. Salmon-coloured columns mean higher cytokine concentrations in patients with CKD than in health controls (adjusted *P*-value <.05). Green-coloured columns mean lower cytokine concentrations in patients with CKD than in healthy controls (adjusted *P*-value <.05).

Patients with CKD had higher plasma levels than healthy controls for IL-5, IL-6, IL-8, IL-17, IL-18, IL-22, chemokine ligand 9 (CXCL9), tumour necrosis factor (TNF)-α, B-cell activating factor (BAFF) and Growth Differentiation Factor 15 (GDF-15) but lower levels of interleukin-10 (IL-10), interleukin-23 (IL-23), chemokine ligand 5 (CCL5), chemokine ligand 17 (CCL17) and TNF-like weak inducer of apoptosis (TWEAK) (Table 2, Fig. 1, Supplementary data, Fig. S3). Additionally, patients with CKD had higher urinary levels of IL-22, chemokine ligand 13 (CXCL13) and GDF-15, and lower urinary levels of IL-8, IL-18 and TNF-α than healthy reference values.

**Table 2:** Baseline cytokine levels in patients with CKD G3 and healthy people.

	CKD G3		Healthy controls			
	Median (Q1–Q3)	*N*	Median (Q1–Q3)	*N*	*P*-value	Adjusted *P*-value
Plasma						
IL-5	5.3 (2.6–10.2)	164	0.1 (0.0–0.5)	28	.000	.000
IL-6	3.2 (2.0–4.6)	163	0.7 (0.5–0.9)	28	.000	.000
IL-8	27.5 (17.8–36.6)	164	5.8 (4.7–7.1)	28	.000	.000
IL-10	3.9 (3.0–5.0)	164	7.3 (6.2–8.8)	28	.000	.000
IL-17	0.2 (0.1–0.4)	164	0.1 (0.1–0.1)	28	.000	.000
IL-18	1007.0 (828.3–1198.4)	163	769.0 (664.2–948.2)	28	.000	.000
IL-22	49.4 (29.1–86.8)	164	11.3 (6.7–19.4)	28	.000	.000
IL-23	2.9 (0.0–31.7)	164	34.0 (0.0–95.0)	28	.008	.011
CCL5	14 697.1 (6550.8–22 876.0)	163	58 515.3 (42 143.5–99 381.6)	28	.000	.000
CCL17	152.2 (102.1–250.5)	163	244.6 (149.0–385.4)	28	.006	.009
CCL20	11.4 (7.1–20.7)	164	12.8 (9.7–17.3)	28	.306	.330
CXCL9	70.8 (44.9–135.7)	164	14.3 (9.4–17.7)	28	.000	.000
CXCL13	59.7 (47.6–80.2)	164	62.7 (49.1–73.5)	28	.830	.862
TNF-α	22.4 (18.4–27.2)	164	8.3 (7.4–9.4)	28	.000	.000
TWEAK	99.6 (81.4–116.5)	164	157.4 (124.2–179.2)	28	.000	.000
BAFF	943.4 (771.8–1095.2)	164	747.3 (692.8–887.0)	26	.000	.000
GDF-15	1865.2 (1108.1–2573.1)	164	481.4 (415.8–566.8)	26	.000	.000
Urine						
IL-6 (U)	1.0 (0.4–3.0)	164	0.5 (0.2–2.1)	26	.187	.210
IL-8 (U)	2.7 (0.0–17.8)	164	13.1 (0.6–68.5)	26	.022	.028
IL-17 (U)	0.0 (0.0–0.0)	164	0.0 (0.0–0.0)	26	.108	.133
IL-18 (U)	0.0 (0.0–5.3)	164	63.2 (44.9–98.6)	26	.000	.000
IL-22 (U)	17.6 (1.9–50.6)	164	0.0 (0.0–7.3)	26	.000	.000
CCL17 (U)	25.6 (12.4–38.6)	163	25.0 (16.2–36.3)	26	.940	.940
CXCL13 (U)	0.0 (0.0–0.4)	164	0.0 (0.0–0.0)	26	.015	.020
TNF-α (U)	0.0 (0.0–0.8)	162	1.3 (0.7–1.8)	26	.000	.000
TWEAK (U)	11.5 (3.9–22.6)	164	12.4 (11.4–26.9)	26	.139	.163
GDF-15 (U)	7902.4 (44 51.5–14 452.2)	163	3364.4 (1711.8–9367.3)	26	.006	.009

Data are presented as median values with interquartile ranges. The *P*-values were calculated using the Mann–Whitney U-test. Adjusted *P*-values were calculated using the Benjamini–Hochberg method.

U, urine biomarker levels.

In general, plasma and urine cytokine levels were aligned for patients with CKD, independently of the presence of T1DM, T2DM or non-DM, although there were some exceptions (Supplementary data, Table S2, Fig. S4). Plasma IL-22, IL-23, CCL5 and CXCL9 were higher in patients with CKD and T2DM than in those without DM. In urine, IL-22 and TWEAK levels were higher in CKD patients with T2DM than in those without DM. These differences in cytokine levels occurred despite differences in baseline clinical characteristics being limited to older age and higher systolic blood pressure values in patients with DM (Supplementary data, Table S3). The number of participants with T1DM was insufficient to draw firm conclusions.

### Individual cytokine levels and baseline characteristics of patients with CKD G3

To investigate the relationship between cytokines and CKD, we first examined the correlation between cytokine levels and baseline eGFR, uACR and HbA1c using Spearman’s rank correlation and multiple regression analyses adjusted for age and sex. In bivariate analysis, plasma TNF-α levels negatively correlated with baseline eGFR (r_s_ = –0.329, *P* < .0001) (Table 3). To test whether these baseline plasma levels are independently associated with clinical parameters after adjustment for age and sex, we performed multivariable regression analyses (Table 4). In bivariate analysis, four plasma cytokines and urinary IL-6 correlated with baseline uACR, but only the correlation between urinary IL-6 (r_s_ = 0.219, *P* = .015) and uACR was confirmed following adjustment for age and sex. Additionally, four plasma cytokines correlated with HbA1C levels but only plasma TNF-α levels were confirmed following adjustment for age and sex.

**Table 3:** Correlation of plasma and urinary cytokines with clinical laboratory parameters (r_s_); bivariate analysis.

	eGFR	eGFR slope	uACR	uACR slope	HbA1c
**r_s_ for correlations with adjusted *P*-value <.1**					
Plasma					
IL-8	NS	NS	0.242	–0.280	NS
IL-10	NS	NS	NS	0.204	NS
IL-22	NS	NS	NS	0.232	0.310
CCL5	NS	NS	0.255	NS	0.350
CCL17	NS	NS	NS	0.161	0.337
CXCL6	NS	NS	NS	0.281	NS
CXCL13	NS	NS	–0.228	NS	NS
GDF-15	NS	NS	NS	0.164	NS
TWEAK	NS	NS	–0.232	NS	NS
TNF-α	–0.326	NS	NS	0.263	0.273
Urine					
IL-6 (U)	NS	–0.338	0.216	NS	NS

Spearman’s rank correlation coefficients between baseline cytokine levels and clinical laboratory parameters. Only significant correlations (Benjamini–Hochberg procedure adjusted *P*-value <0.1) are displayed in the table. Cytokines that showed no statistically significant correlation with any of the clinical laboratory parameters are not shown.

U, urine biomarker levels; NS, nonsignificant.

**Table 4:** Correlation of plasma and urinary cytokines with clinical laboratory parameters (*P*-value); multiple regression analysis.

	eGFR	eGFR slope	uACR	uACR slope	HbA1c
***P*-value**					
Plasma					
IL-8	NS	NS	NS	0.001	NS
IL-17	NS	NS	NS	NS	0.002
IL-18	NS	NS	NS	NS	0.025
IL-22	NS	NS	NS	0.000	NS
CXCL13	NS	NS	NS	0.000	NS
GDF-15	NS	0.010	NS	0.017	NS
TNF-α	0.002	NS	NS	NS	0.001
BAFF	NS	NS	0.007	NS	0.027
Urine					
IL-6 (U)	NS	0.006	0.000	NS	0.032
IL-8 (U)	NS	0.043	NS	NS	NS
IL-17 (U)	NS	NS	0.000	NS	NS
IL-22 (U)	NS	0.031	NS	NS	NS
CCL17 (U)	NS	NS	NS	NS	0.010
TWEAK (U)	0.016	NS	NS	NS	NS

Multiple regression analysis for baseline cytokine levels and clinical laboratory parameters, adjusted for age and sex. Only significant correlations between cytokine levels and clinical parameters are displayed in the table. Cytokines that showed no statistically significant correlation with any of the clinical laboratory parameters are not shown.

U, urine biomarker levels; NS, nonsignificant.

### Individual cytokine levels and outcomes of patients with CKD G3

Next, we evaluated correlations between baseline cytokine levels and short-term (36 months) kidney outcomes defined as uACR and eGFR slopes. In bivariate analysis, urinary IL-6 levels negatively correlated with eGFR slope (r_s_ = –0.338, *P* < .0001) (Table 3) and this correlation was confirmed following adjustment for age and sex (Table 4). In bivariate analysis, seven plasma cytokines also correlated with uACR slope, but only the correlation between plasma IL-8, IL-22, CXCL13 and GDF-15 was confirmed following adjustment for age and sex. HbA1c levels were significantly correlated with IL-22, CCL5, CCL17 and TNF-α.

The need for KRT and mortality was examined at 10 years of follow-up (Table 1). Overall, 21 (20.6%) patients started KRT and 54 (34.4%) of 157 patients with information available died. The combined outcome of KRT or death was observed in 75 (47.8%) patients.

To test whether baseline plasma levels of the cytokines that showed significant associations with uACR or eGFR deterioration (IL-22, TNF-α, IL-8 and GDF-15) were independently associated with the outcome after adjustment for age and sex, we performed a multivariable logistic regression analysis. Plasma GDF-15 levels were significantly associated with KRT or death and with death, while both plasma GDF-15 and TNF-α were significantly associated with KRT (Table 5).

**Table 5:** Multivariate association with outcomes.

	Estimate	Std error	z value	Pr(>|z|)
(A) Multiple logistic analysis for death or KRT				
(Intercept)	–4.30E+00	1.39E+00	–3.092	1.99E-03
Age	2.67E−02	1.86E−02	1.438	0.15053
Sex	–2.16E−01	4.26E−01	–0.507	0.61242
IL-22	–4.97E−04	1.37E−03	–0.364	0.71600
TNF-α	4.59E−02	2.40E−02	1.916	0.05534
IL-8	1.05E−03	1.18E−02	0.089	0.92933
GDF-15	8.18E−04	2.28E−04	3.583	0.00034
(B) Multiple logistic analysis for death				
(Intercept)	–1.26E+01	2.51E+00	–5.01	5.44E−07
Age	1.41E−01	3.24E−02	4.355	1.33E−05
Sex	–7.16E−01	5.51E−01	–1.3	0.19346
IL-22	1.12E−03	1.52E−03	0.739	0.45977
TNF-α	2.66E−02	2.28E−02	1.167	0.24325
IL-8	3.14E−03	1.30E−02	0.242	0.80853
GDF-15	6.82E−04	2.23E−04	3.053	0.00227
(C) Multiple logistic analysis for KRT				
(Intercept)	–1.2654948	1.5745904	–0.804	0.42157
Age	–0.041446	0.0224432	–1.847	0.06479
Sex	0.0936797	0.5295498	0.177	0.85958
IL-22	–0.0016023	0.0022135	–0.724	0.46913
TNF-α	0.0656064	0.0292932	2.24	0.02511
IL-8	–0.0020795	0.0162488	–0.128	0.89816
GDF-15	0.0008266	0.0003069	2.693	0.00707

### Stratification based on unsupervised cluster analysis of plasma cytokine levels

**Figure 2:**
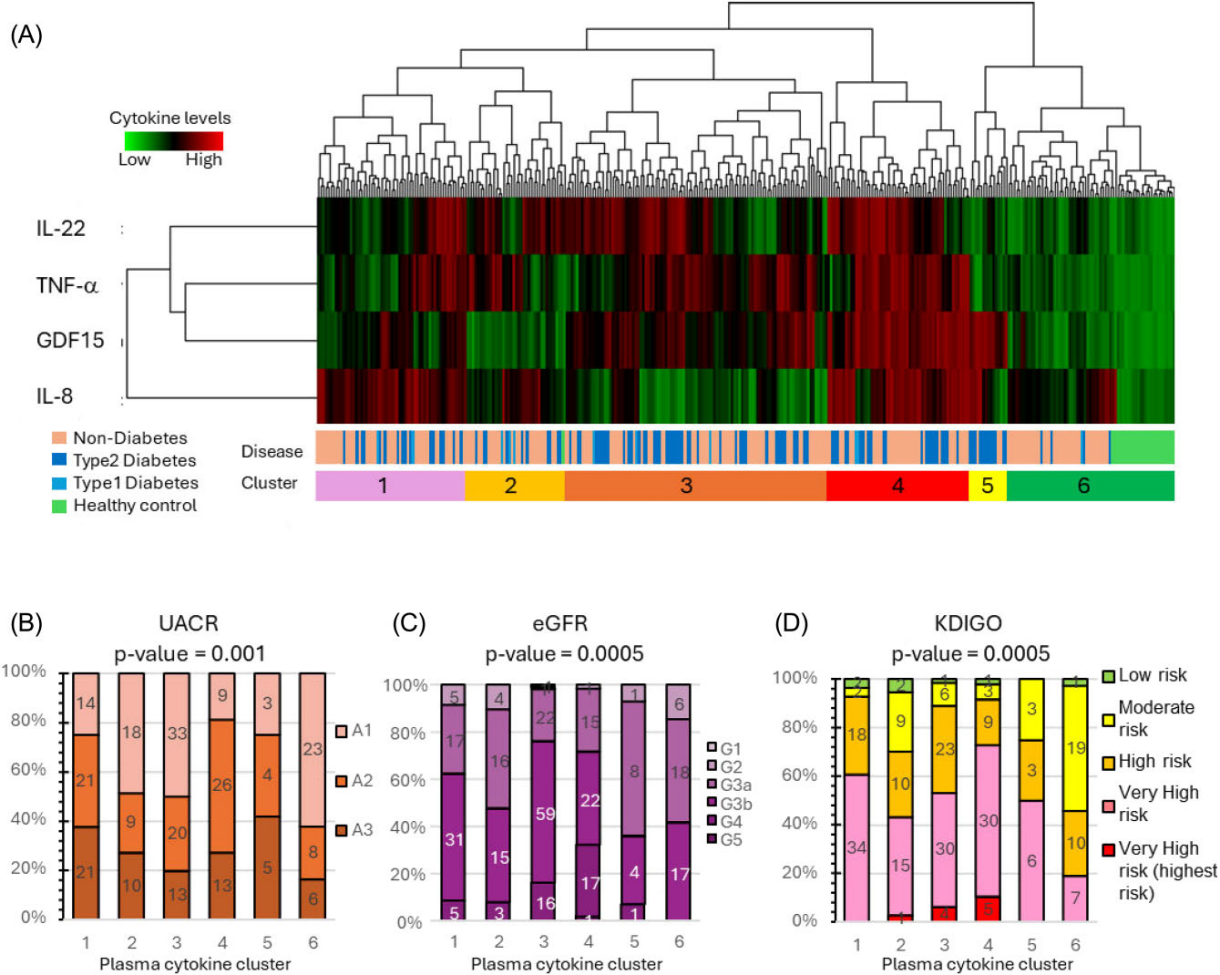
Cluster analysis of plasma cytokine levels. Cluster analysis was performed for all sample points of the plasma cytokines that showed both higher baseline levels in patients with CKD than in healthy controls and a significant correlation with baseline eGFR or uACR slopes in both univariate and multivariate analyses: IL-22, TNF-α, GDF-15 and IL-8. (**A**) Cluster analysis identified six cytokine-based clusters, shown at the bottom. Cluster 6 was characterized by low plasma levels of all four cytokines and contained most healthy controls and some CKD patients who, for the most part, did not have DM. By contrast, Cluster 6 was characterized by high levels of all four plasma cytokines and contained mostly patients with CKD as indicated at the bottom. (**B**–**D**) Association of the plasma cytokine clusters with uACR (B), eGFR (C), and KDIGO risk categories (derived from the combination of uACR and eGFR values) (D). In (B) to (D) all available data points are represented. Statistical significance between the clusters was calculated using Fisher’s exact test.

Plasma IL-8, IL-22, CXCL13, TNF-α, GDF-15 and urinary IL-6 demonstrated significant correlations with baseline or future eGFR or uACR values in both univariable and multiple regression analyses and GDF-15 was also associated with mortality. Among these, plasma IL-8, IL-22, TNF-α and GDF-15 levels were significantly higher in CKD patients compared with healthy reference values, suggesting their potential role as key inflammatory mediators in CKD progression (Table 1). Cluster analysis was performed using the plasma levels of these four cytokines at baseline, 18 months and 36 months to examine cytokine patterns (Fig. 2A, Supplementary data, Fig. S5) and associations with kidney, cardiovascular and survival outcomes. In this analysis, patients were grouped according to shared patterns of cytokine levels. Unsupervised cluster analysis classified plasma cytokine profiles into six clusters: Cluster 1 (IL-8 High), Cluster 2 (IL-8 High, GDF-15 Low, IL-22 High), Cluster 3 (TNF-α High, GDF-15 High, IL-22 Partially High), Cluster 4 (All High), Cluster 5 (GDF-15 High) and Cluster 6 (All Low). Most healthy individuals were classified into Cluster 6.

Plasma cytokine clusters were associated with uACR, eGFR and KDIGO risk categories in a statistically significant manner among patients with CKD (Fig. 2B–D). In Cluster 6, 60% of samples were categorized as A1 based on uACR criteria (Fig. 2B), 41% were G3a or lower (Fig. 2C) and more than half were classified as moderate risk or lower according to KDIGO risk categories (Fig. 2D). In contrast, Cluster 4 (All High) exhibited the most severe disease phenotype, with 80% of patients classified as A2 or higher (Fig. 2B), 26.7% were G3b or higher (Fig. 2C) and over 70% categorized as Very High KDIGO risk at 36 months (Fig. 2D). Clusters 1 and 4 (high plasma IL-8) had the higher prevalence of high albuminuria A categories and high KDIGO risk categories while Clusters 3 and 4 (high plasma TNF-α, GDF-15, IL-22) had the highest prevalence of more advanced eGFR G categories. Cluster 6 (All Low) had the mildest disease phenotype, representing patients with slower disease progression.

Time-series analysis of cluster assignments showed that half of the patients in Clusters 1 and 2 transitioned to other clusters, with frequent movement between Clusters 1 and 2 compared with transitions involving other clusters. Patients in Clusters 3 and 4 remained in their high-risk groups throughout the study period. Cluster 6 exhibited the most stability, but 42% of patients transitioned to Cluster 1 or Cluster 3 (Table S4). Overall, there was a pattern of transition to clusters associated with more severe CKD over time. In this regard, clusters representing the most severe inflammation (3 and 4: High TNF-α, GDF-15, IL-22) were the most stable over time and a common destination of patients transitioning from Cluster 1 and 6 to other clusters. Detailed data on baseline cytokine levels in each cluster are presented in Supplementary data, Fig. S6.

### Association of unsupervised clusters of plasma cytokine with DM, baseline kidney function and short-term kidney function and cardiovascular outcomes

**Figure 3:**
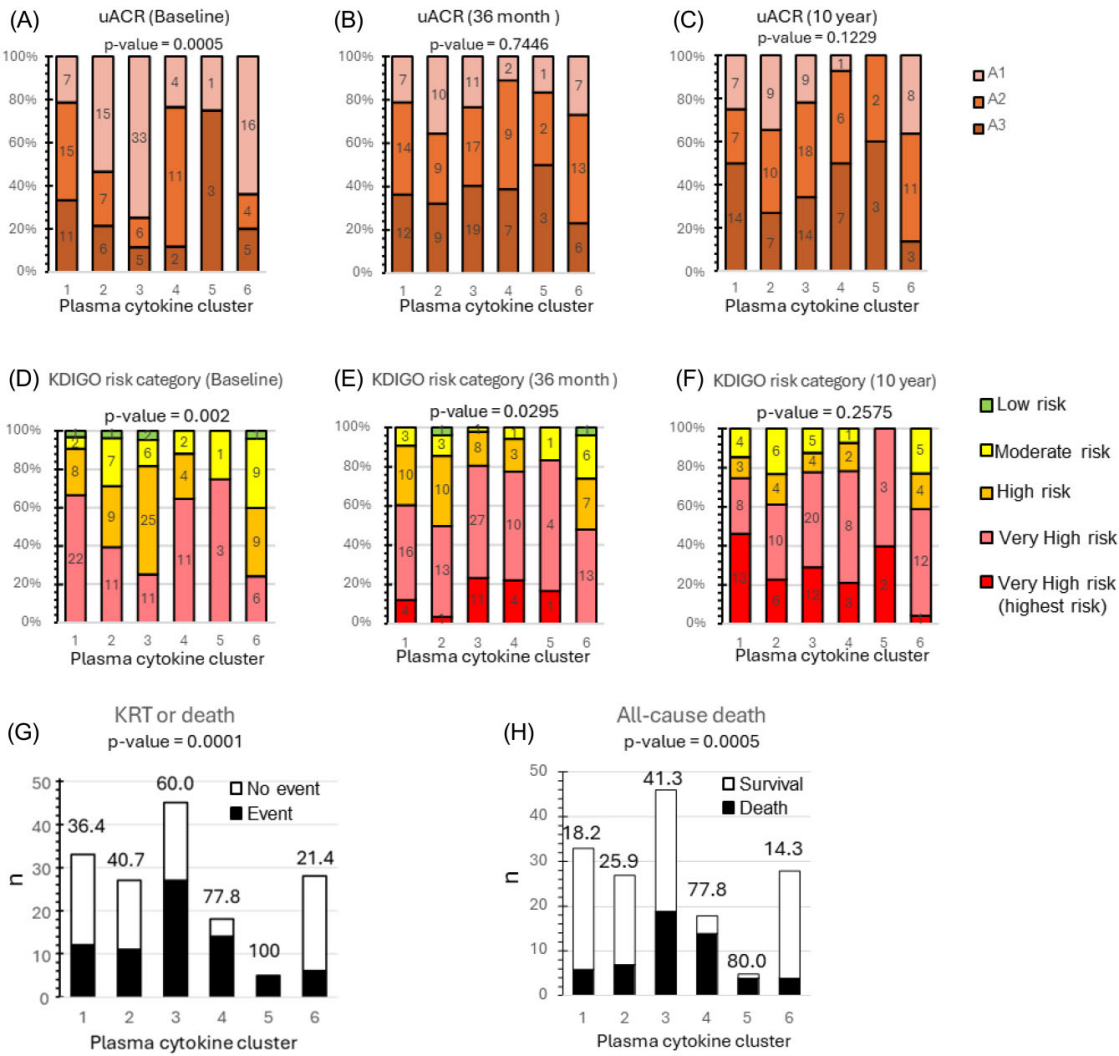
Kidney baseline and outcome features and long-term mortality data associated with baseline plasma cytokine clusters. (**A**–**F**) uACR (A–C) and KDIGO risk class (D–F) at baseline (A, D), 36 months (B, E) and 10 years (C, F) for the different baseline plasma cytokine clusters. The vertical axis represents the percentage of patients in each category and the number within the segments of the columns indicates the number of patients in each category. (**G**) KRT (dialysis or kidney transplantation) within 10 years according to different baseline plasma cytokine clusters. (**H**) Mortality within 10 years according to different baseline plasma cytokine clusters. In (G) and (H), the number on top of each column represents percentage of patients with event. Statistical significance among the clusters was calculated using Fisher’s exact test.

Overall, baseline plasma cytokine clusters differed in age, prevalence of DM and most measures of kidney function and uACR either as baseline or as outcomes (Fig. 3, Supplementary data, Fig. S7, Table S5).

Compared with an overall prevalence of DM of 39.4% (Table 1), the prevalence of DM differed in a statistically significant manner between plasma cytokine clusters, from 13.7% in Cluster 6 to 50% in Clusters 3 and 4, with Cluster 5 showing the highest proportion (83.3%) (Supplementary data, Fig. S7A).

In the whole CKD group, median eGFR decreased from 42.8 to 39.5 mL/min/1.73 m^2^ at 36 months and 31.7 mL/min/1.73 m^2^ at 10 years (Table 1). The annualized eGFR slope up 36 months was –1.2 mL/min/1.73 m²/year. The median baseline uACR of 42.7 mg/g had increased to 113.5 mg/g at 36 months, and 111.5 mg/g at 10 years. The annualized uACR increase was 11.7 mg/g/year.

Patients in Cluster 6, characterized by low cytokine levels, were generally younger and had better preserved baseline eGFR and lower uACR values, and also lower serum creatinine and urea levels compared with other clusters at both baseline and 36 months, in some cases reaching statistical significance, such as the difference for baseline and 36-month eGFR, urea and age versus Cluster 3 (Supplementary data, Fig. S7B, C, N–P, Table S5).

Cluster 3 was characterized by elevated levels of multiple cytokines—including plasma TNF-α, IL-22, GDF-15, IL-18, IL-23 and CXCL9, but not plasma IL-8 (Fig. 2A, Supplementary data, Fig. S6). Indeed, Cluster 3 had significantly worse outcomes than Cluster 6 as reflected by larger delta eGFR and faster annualized eGFR and uACR slopes (Supplementary data, Fig. S7E, F, K) estimated from baseline to time 36 months (Supplementary data, Table S5).

When assessing albuminuria categories A1 through A3, there were statistically significant differences between plasma cytokine clusters at baseline (Fig. 3A) but not during follow-up (Fig. 3B and C). Clusters 3 and 6 had the lowest prevalence of A2–A3 uACR at baseline (Fig. 3A). This contributed also having the lowest baseline prevalence of the very high risk KDIGO category (Fig. 3D), as was also the case at 36 months, at which time point, Clusters 3–5 had the highest prevalence of very high risk KDIGO category (Fig. 3E). However, by 10 years of follow-up, very high risk was the most common status for all clusters and there were no statistical significant differences (Fig. 3F).

Cluster 4 exhibited the highest elevation in multiple inflammatory cytokines (Fig. 2A, Supplementary data, Fig. S6). Like Cluster 3, it also displayed some statistically significant differences with Cluster 6, such as older age and higher baseline and 36 months urea levels (Supplementary data, Fig. S7N–P). The uACR outcomes were also worse than in Cluster 6, as judged by significantly higher uACR levels at 10 years (Supplementary data, Fig. S7I, Table S5). Approximately 10% of patients had uACR A3 at baseline, increasing to 40% at 36 months and 50% at 10 years (Fig. 3A–C). Over 60% of patients were classified as Very High on the KDIGO risk score at baseline, rising to nearly 80% at 36 months and 85% at 10 years, with 20% of these classified as Very High (highest risk) at 36 months and 10 years (Fig. 3D–F).

Plasma cytokine clusters were also associated with CVD progression assessed as cardiovascular hospitalizations within 36 months. These were less common for Cluster 6 and more common for Clusters 3–5 (Supplementary data, Fig. S8A) (*P* = .025).

### Plasma cytokines and long-term KRT and mortality outcomes

At 10 years, death was a more common outcome than KRT (Table 1). Baseline plasma cytokine clusters were associated with the primary outcome of death or KRT, which occurred in 20.4% of participants, with a range of 21.4%–100% in different clusters, being more frequent in Clusters 3–5 (*P*-value = .0001) (Fig. 3G). Mortality rates also significantly differed across plasma cytokine clusters, ranging from 14.3% (Cluster 6) to 77.8% (Cluster 4) and 80.0% (Cluster 5) (Fig. 3H) (*P* = .0005). A similar trend was observed for KRT. Within 10 years of follow-up, the incidence of KRT was 20.4% overall. Across plasma cytokine clusters, it ranged from 0.0% (Cluster 4) to 33.3% (Cluster 3) and 100.0% (Cluster 5) (Supplementary data, Fig. S8B) (*P* = .1128). It is likely that the high mortality in Clusters 4 and 5 competed with the KRT outcome.

## DISCUSSION

In our study of CKD G3 patients, plasma cytokine clusters characterized by high TNF-α and GDF-15 (Clusters 3, 4 and 5) showed markedly higher risks of kidney failure and mortality at 10 years (up to 80% mortality in Cluster 5), whereas Cluster 6, with low cytokine levels, was associated with preserved kidney function and the lowest risk of adverse outcomes. These observations demonstrate a direct association between systemic inflammatory profiles and both disease severity and long-term prognosis. This information may be used to characterize novel molecular mechanisms involved in CKD progression and in the systemic impact of CKD. In this regard, while most plasma cytokines were dysregulated, only 10 (60%) were upregulated in the plasma of CKD patients, presenting a complex picture of cytokine dysregulation which differed from a widespread increase in plasma cytokine levels. These cytokines and their plasma cytokine clusters may be used for risk stratification and, potentially, to assess the response to therapy.

This study provides further evidence supporting the critical role of systemic inflammation in CKD progression, demonstrating significant differences in cytokine levels between CKD patients and healthy individuals, irrespective of diabetes status, and the association of plasma cytokine levels with outcomes. Notably, urinary cytokine elevations were more limited. These findings suggest that systemic inflammation, rather than localized kidney inflammation, plays a dominant role in CKD pathophysiology and outcomes. Overall, systemic inflammation appeared to be more prominent in patients with DM, in line with the literature [10]. When compared with prior studies using similar methods, the present study is consistent with differences in cytokine patterns between primary and secondary nephropathies. In primary glomerular diseases such as primary membranous nephropathy, urinary TWEAK and IL-18 levels are markedly elevated but these cytokines were not detected in CKD patients within the present cohort. Conversely, urinary IL-22, absent in primary membranous nephropathy, was elevated in T2DM-associated CKD, further reinforcing the distinct inflammatory profiles of at least some nephropathies secondary to systemic disease [13]. Meanwhile, autoimmune kidney diseases such as lupus nephritis involve distinct inflammatory pathways, including IL-4-driven type 2 immune responses [14, 15].

After adjusting for confounders such as age and sex using multiple regression analysis, significant correlations were observed between plasma levels of IL-8, IL-22, GDF-15 or TNF-α or urine IL-6 and kidney function in patients with CKD G3. However, the correlation coefficients for individual cytokines were consistently weak. As a next step, cluster analysis was performed using plasma levels of the four cytokines that were both significantly elevated in CKD patients and correlated with kidney function. TNF-α is a pro-inflammatory cytokine that has been implicated in the pathogenesis of acute kidney injury and CKD and its receptors are associated with adverse outcomes in diabetic kidney disease [16–20]. GDF-15 is increased in plasma and urine of people with CKD and is associated with adverse outcomes [21–25], despite having a direct kidney protective effect [26, 27]. IL-22 and IL-8 findings are more novel. IL-22 belongs to the IL-10 family and is primarily produced by T cells [such as T helper (Th) 22 and Th17] and innate immune cells such as ILC3 [28]. In systemic lupus erythematosus, plasma IL-22 levels correlate with the severity score and with decreased kidney function [29]. IL-8 is a CXC chemokine encoded by the *CXCL8* gene that facilitates the migration of inflammatory cells such as neutrophils [30, 31]. Plasma IL-8 levels increase in kidney failure, while urinary IL-8 levels negatively correlated with eGFR in children with CKD G2–G4 [32, 33]. In this regard, the *CXCL8* gene is constitutively expressed by glomerular parietal epithelial cells [34, 35].

A key feature of the present study is the use of unsupervised cluster analysis to stratify patients into clusters based on their inflammatory profiles. Overall, plasma cytokine clusters representing more severe systemic inflammation were associated with more severe CKD or more rapid CKD progression, and worse kidney and patient survival outcomes (primary outcome). In contrast, clusters with lower systemic inflammation tended to exhibit relatively slower disease progression. From a clinical perspective, our results emphasize the potential utility of cytokine profiling in risk stratification. The identification of distinct inflammatory phenotypes raises the possibility of using biomarker-based approaches to predict CKD progression and tailor therapeutic strategies accordingly.

Plasma cytokine Clusters 3 through 5 were associated with adverse long-term hard outcomes, such as death and KRT. Beyond the four cytokines used to build the clusters, clusters were associated with wider cytokine pattens. For example, Cluster 3 exhibited high plasma levels of TNF-α, GDF-15, IL-22, IL-18, IL-23, CCL17, CCL20 and CXCL9, while IL-8 levels were low. Cluster 4 presents the most significant elevation in plasma cytokine levels. Cluster 5 was uncommon and characterized by high plasma levels of GDF-15 and CXCL9, with 75% of patients having a KDIGO risk score of Very High at baseline. Within 10 years, 80% of Cluster 5 patients had died. However, due to the small sample size, definite conclusions cannot be drawn for Cluster 5. Cluster 2 represented the mildest systemic inflammation after Cluster 6. In Cluster 2, no significant changes in uACR severity or KDIGO risk score were observed from baseline to 36 months and mortality within 10 years was 20%.

Cytokine clusters also provided preliminary information into potential inflammatory cytokine journeys. People in Cluster 1 tended to transition to Clusters 3 or 4. If confirmed, this information may allow identification of patients in early stages of systemic inflammation and offer them participation in trials of anti-inflammatory interventions.

By contrast to Clusters 3–5, plasma cytokine Cluster 6 exhibited the lowest levels of systemic inflammation and was associated with milder disease as assessed by several baseline and outcome parameters, suggesting that this group comprises individuals with slow disease progression.

Several limitations should be noted, including those inherent to observational studies. First, although the PROGRESER study initially enrolled 462 patients, this substudy was restricted to the 165 individuals for whom biobanked plasma and urine samples were available. This reduced sample size may have limited the statistical power to detect significant differences and may affect the generalizability of the findings. Second, while the primary composite outcome was KRT or death, no data were collected on the temporal sequence of these events, precluding analysis of whether KRT preceded mortality. Third, healthy individuals providing reference values were younger than the CKD population, which may have influenced comparisons of cytokine profiles. However, our primary outcomes were based on analyses within the CKD cohort, and all multivariable models were adjusted for age, thereby minimizing the impact of this difference.

Nevertheless, the study also has strengths. It is widely representative of real-world patients with CKD G3 under nephrology care, enrolling participants from 14 of 17 regions in Spain. The extended follow-up of ten years allowed the assessment of hard outcomes. Finally, as uptake of novel kidney protective drugs, such as sodium-glucose cotransporter 2 (SGLT2) inhibitors and glucagon-like peptide 1 receptor (GLP-1R) agonists, increases, the present cohort may serve as a comparator for future studies that assess the impact of novel drugs over the prior standard for kidney protection.

In conclusion, specific plasma cytokine patterns consistent with systemic inflammation are associated with baseline CKD severity in patients with CKD G3, as well as with short-term kidney outcomes and long-term hard outcomes. Specific plasma cytokine clusters consisting of four cytokines helped to condense the information contained in a larger cytokine panel and may inform research into novel pathophysiological pathways as well as risk stratification and response to therapy. These clusters may help design clinical trials of anti-inflammatory therapies targeting specific inflammatory cytokines. Among individual cytokines, plasma GDF-15 emerged as a particularly strong predictor of long-term outcomes. Multivariable logistic regression analysis identified age and plasma GDF-15 levels as independent predictors of 10-year mortality, underscoring its potential role as a prognostic marker.

## Supplementary Material

sfaf352_Supplemental_Files

## Data Availability

The data underlying this article will be shared on reasonable request to the corresponding author.
