# Supplementary material for: Systemic inflammatory profiles are associated with long-term kidney failure and patient mortality in chronic kidney disease
Source: Clin Kidney J. 2025 Dec 9;19(4):sfaf352. doi: 10.1093/ckj/sfaf352 (PMC13053082; doi:10.1093/ckj/sfaf352)
Supplement: sfaf352_Supplemental_Files [file sfaf352_supplemental_files.zip › SUPPL Figure progresser june 13 .pdf]

Figure S1. Study design and outcome definition. Outcomes indicated in blue (up to 36 months) and red (up to 10 years)

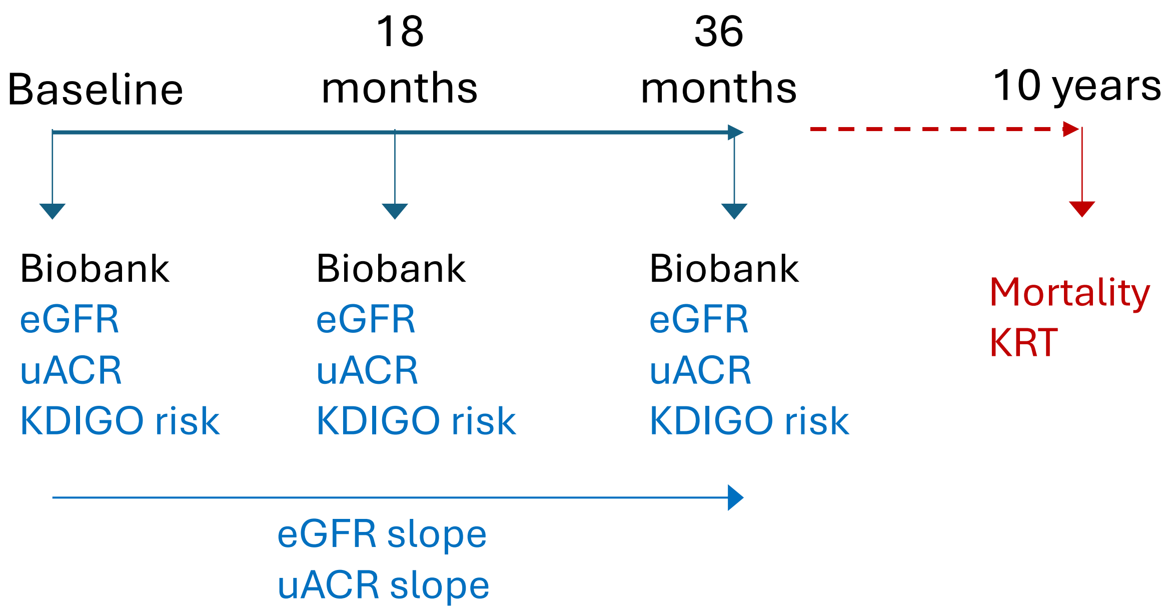

Figure S2. KDIGO risk categories based on eGFR G categories and albuminuria A categories (KDIGO 2012 Clinical Practice Guideline for the Evaluation and Management of Chronic Kidney Disease. Kidney Int. 2013, 3, 5–14). Patients included in the study are delineated by the bold dashed box within the KDIGO heat map

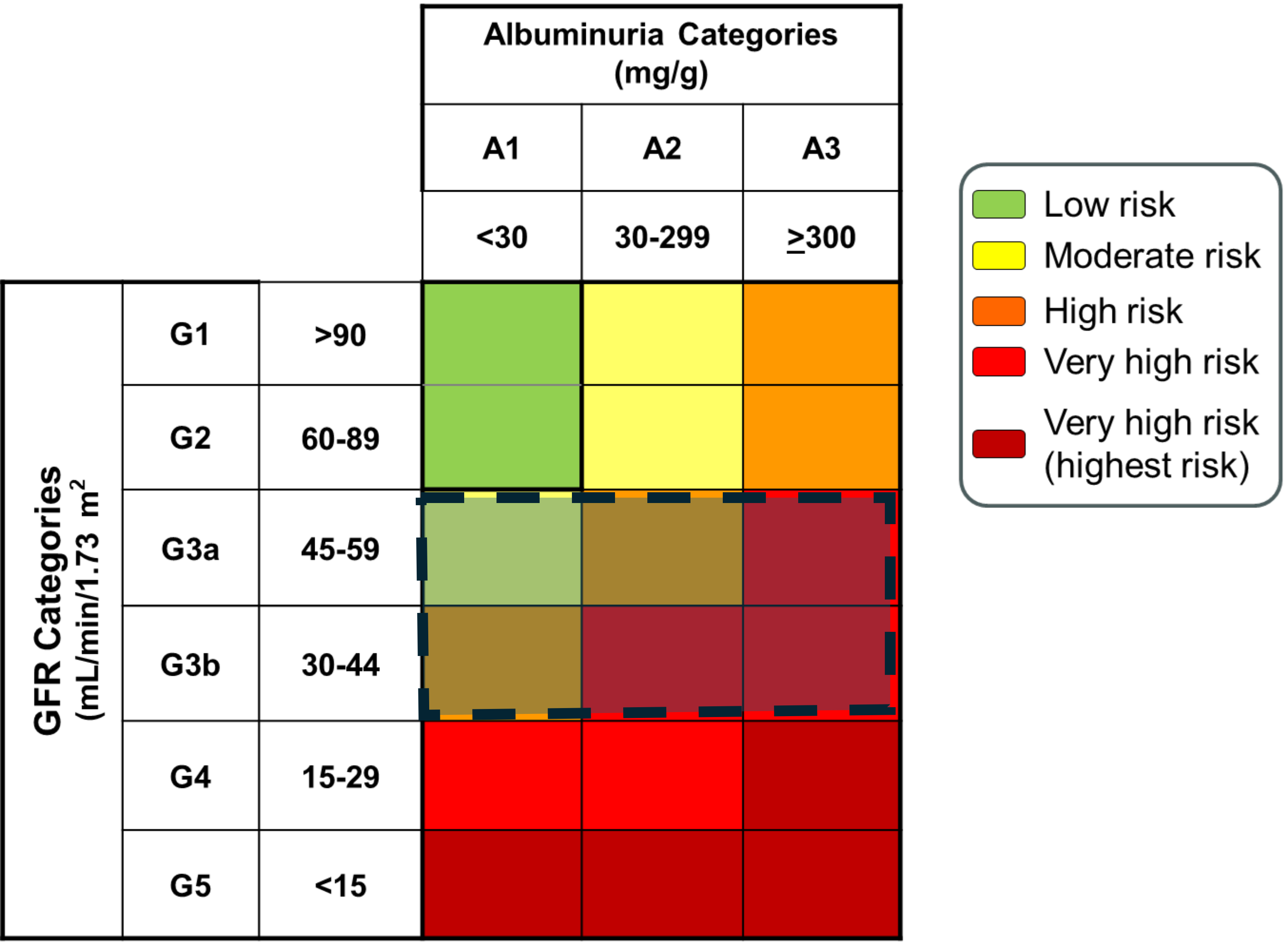

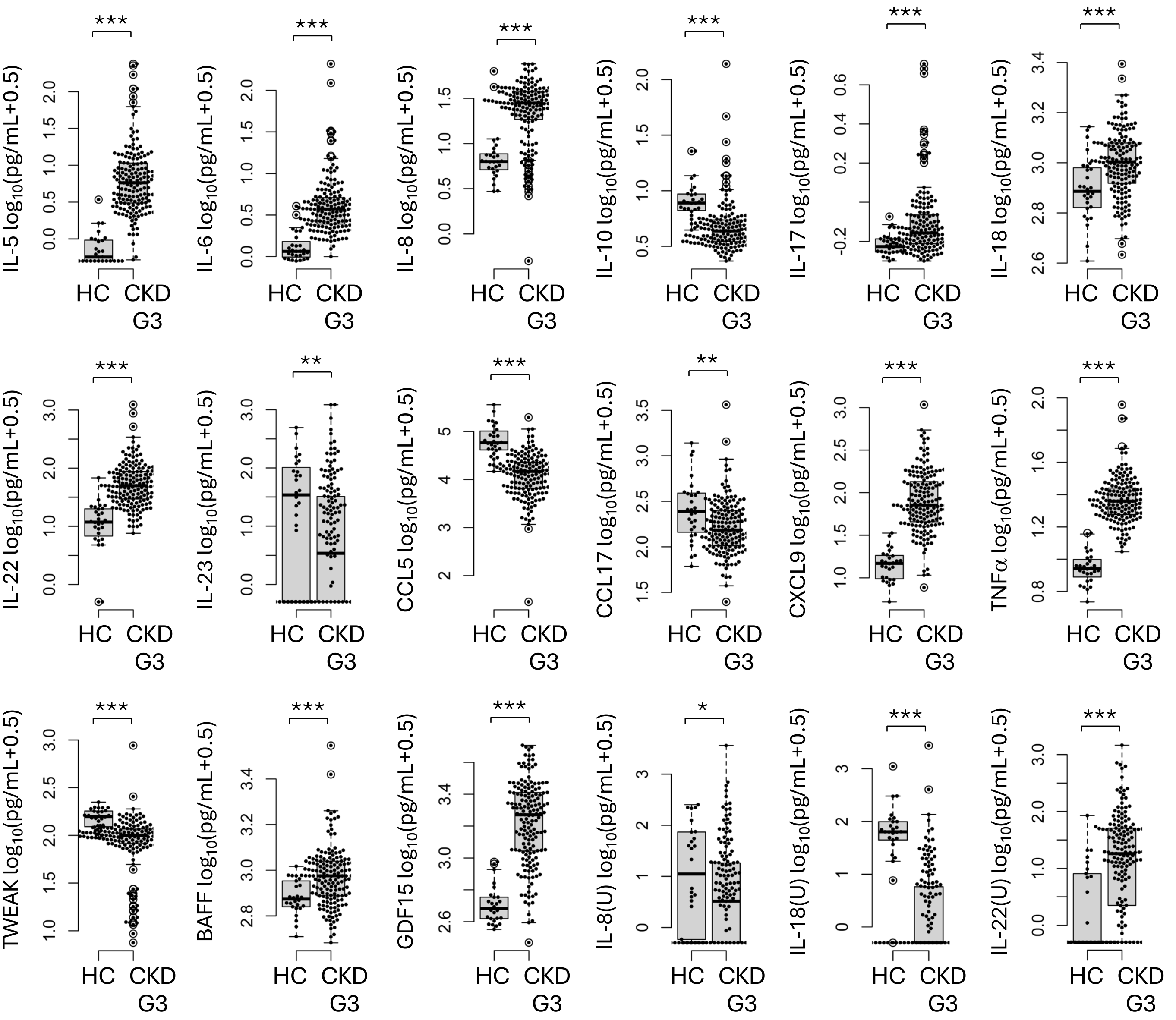

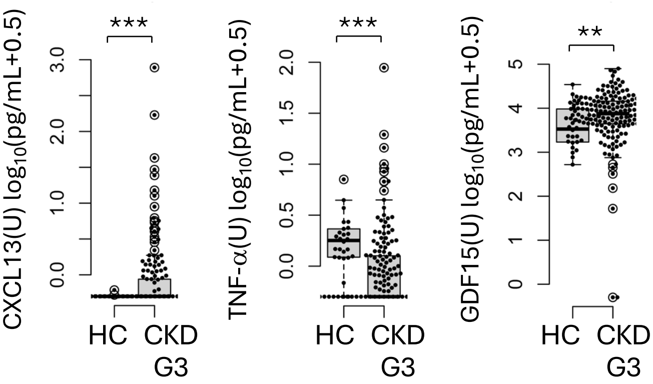

Fig. S3. Cytokine levels in patients with CKD G3 and healthy controls (HC) .
The results are shown as individual data points with a median (bar) and an interquartile range (box). U: urine biomarker levels. P-values were calculated using the Mann-Whitney U-Test, *: $P < 0.05$, **: $P < 0.01$, ***: $P < 0.001$.

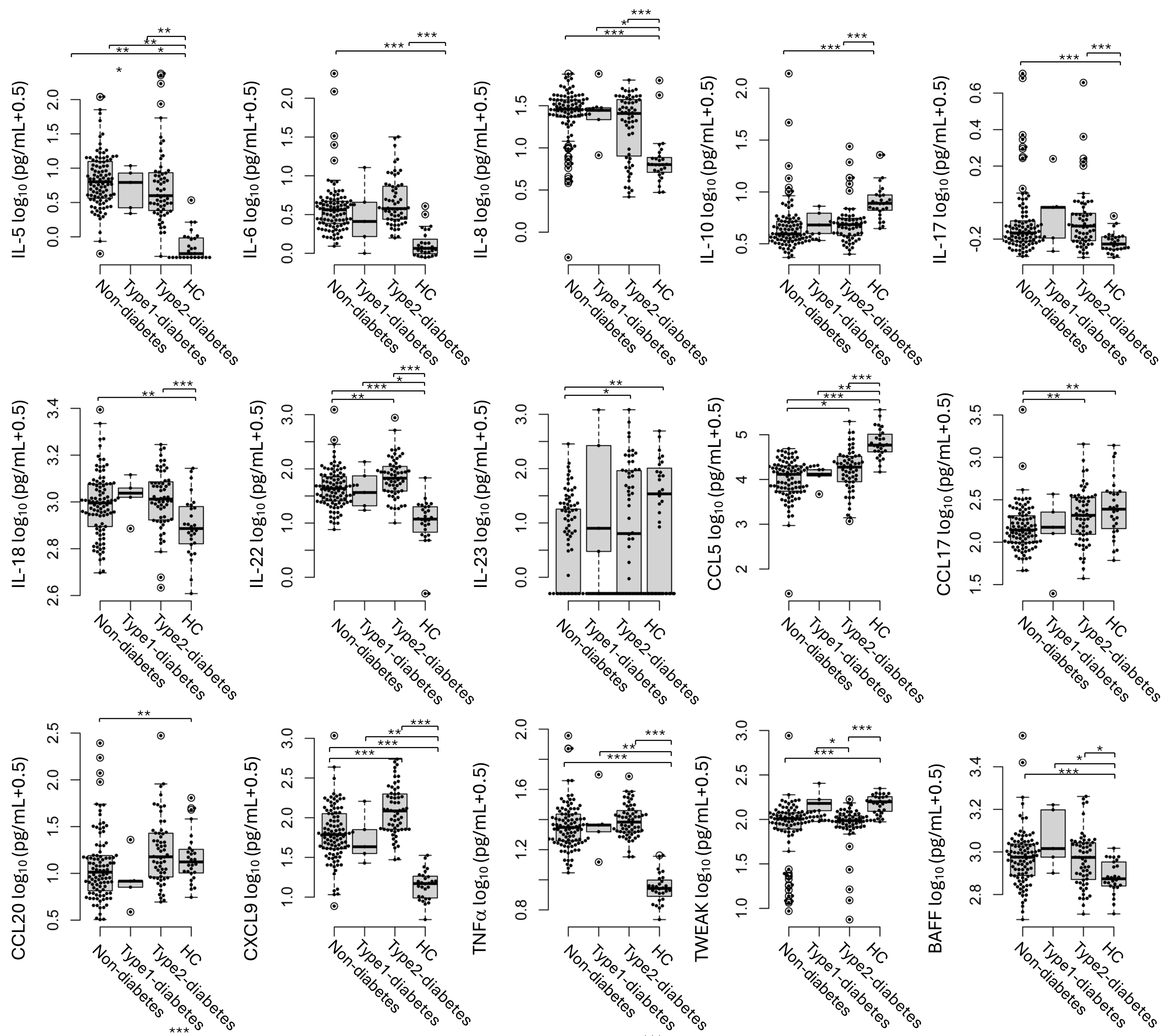

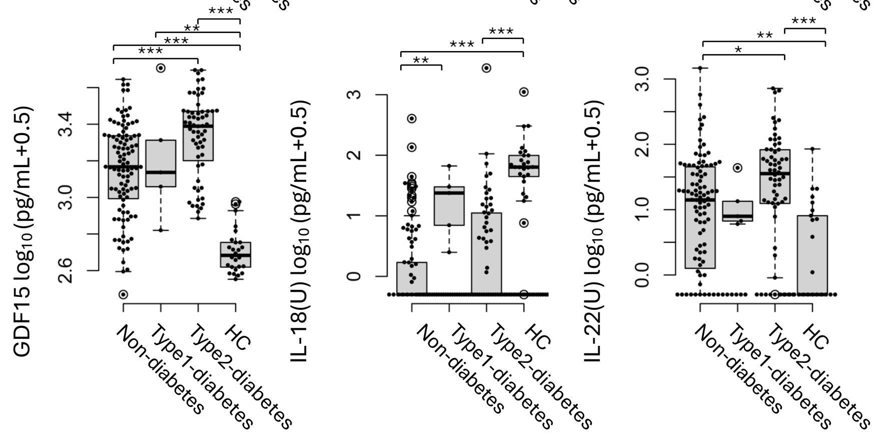

Fig. S4. Cytokine levels in subgroups according to presence of DM among patients with CKD G3.
The results are shown as individual data points with a median (bar) and an interquartile range (box). U: urine biomarker levels. P-values were calculated using the Steel–Dwass test. *: $P < 0.05$, **: $P < 0.01$, ***: $P < 0.001$.

A

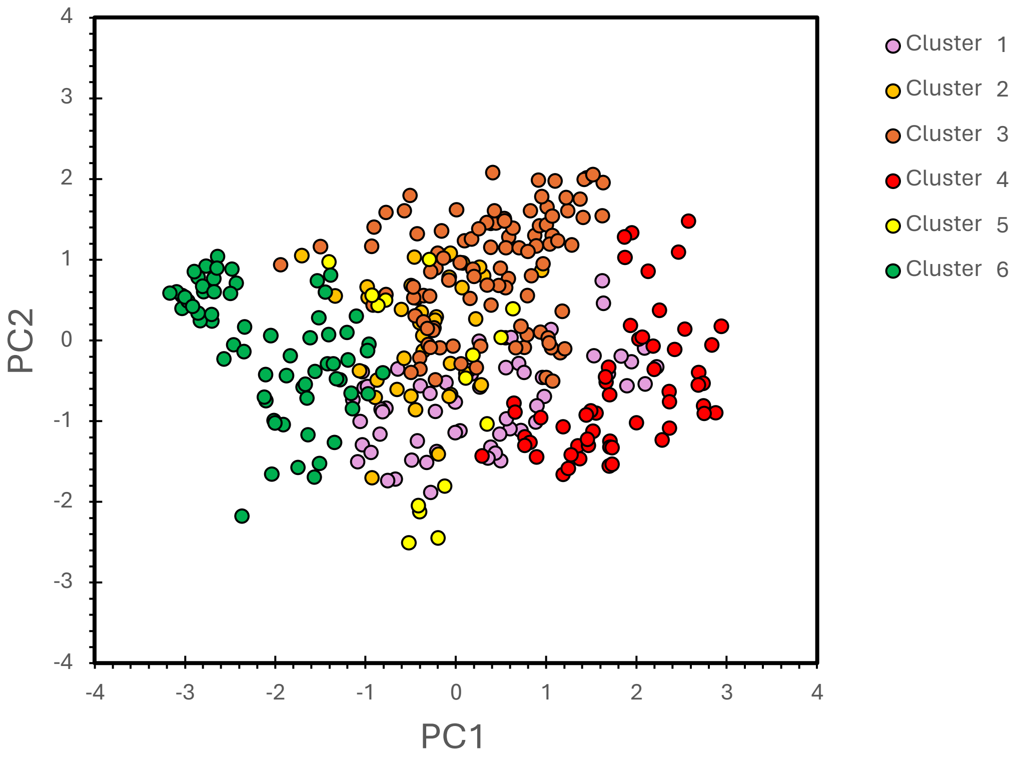

B

	PC1	PC2	PC3	PC4
IL-22	0.289	0.069	0.563	0.079
TNF-α	0.325	0.057	0.044	0.574
IL-8	0.062	0.868	0.043	0.026
GDF15	0.323	0.006	0.35	0.321

Fig. S5. Principal component analysis (PCA) of plasma levels of IL-8, IL-22, TNF-α, and GDF15. A) Scatter plot of principal components 1 and 2, with clusters indicated by color. B) Explained variance ratio for each principal component.

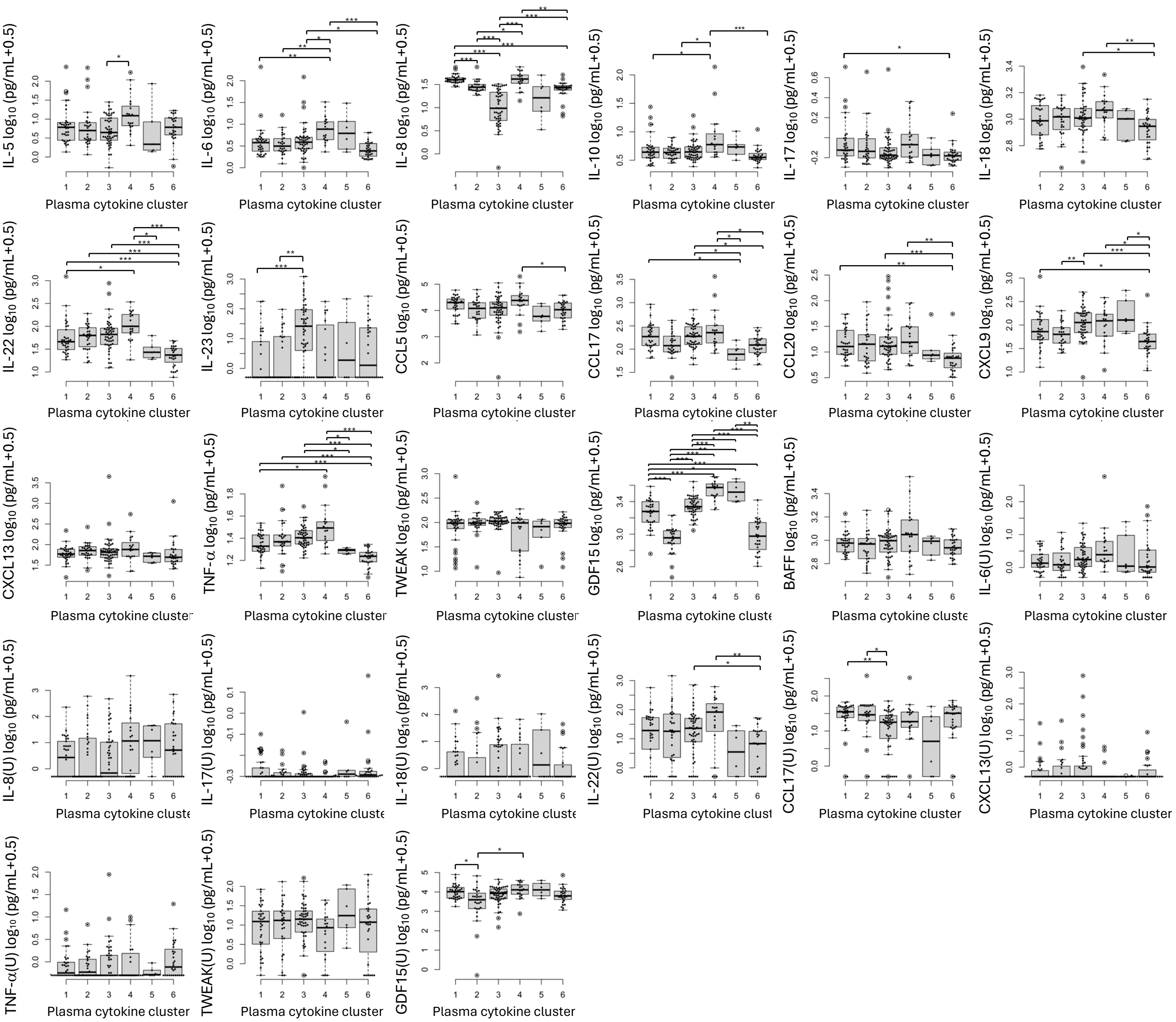

Fig. S6. Baseline levels of cytokines for each cytokine cluster defined by plasma levels of IL-8, IL-22, TNF-α, and GDF15. Clusters are indicated at the bottom. The results are displayed as individual data points, with the median represented by a bar and the interquartile range shown as a box. U: urinary biomarker levels. P-values were calculated using the Steel–Dwass test: * P < 0.05, ** P < 0.01, *** P < 0.001.

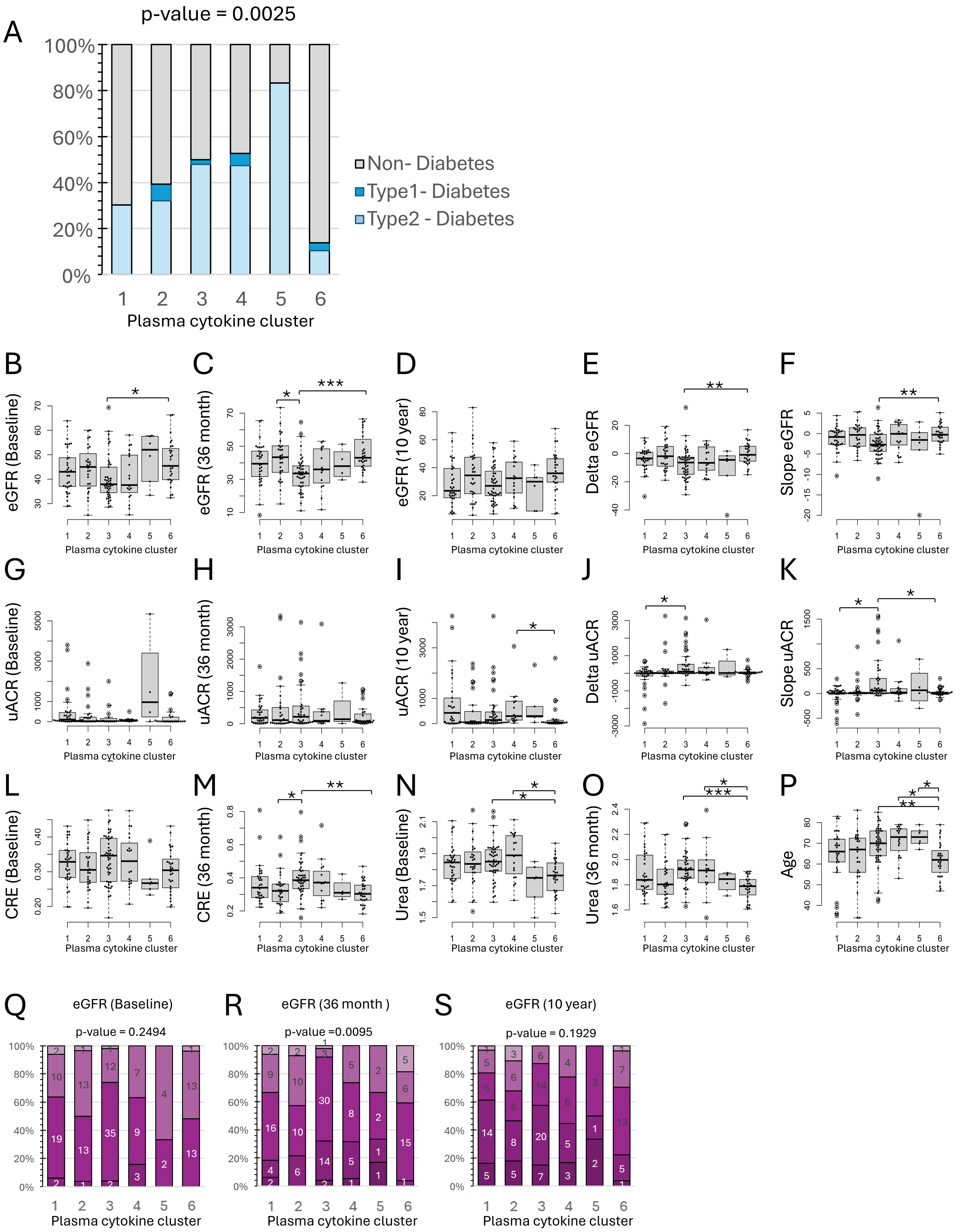

Fig. S7. Diabetes mellitus (DM) and baseline and outcome kidney-related analytical values associated with the plasma cytokine clusters. A) Type 1 DM, type 2 DM and non-DM. **B-P**) eGFR (**B-F**), UACR (**G-K**), serum creatinine (**L-M**), serum urea (**N-O**), and age (**P**) values are shown for the different clusters at baseline (**B, F, L, N)** and during follow-up as well as delta values for eGFR (**E**) and UACR **(J)** and annualized slopes FOR eGFR (**F**) and UACR (**K**) estimated from baseline to 36 month. The results are displayed as individual data points, with the median represented by a bar and the interquartile range shown as a box. U: urinary biomarker levels. P-values were calculated using the Steel–Dwass test: * P < 0.05, ** P < 0.01, *** P < 0.001.**Q-S**) eGFR values at baseline (**Q**), 36 months (**R**), and 10 years (**S**) for the different clusters. Statistical significance among the clusters was calculated using Fisher's exact test. Clusters are indicated at the bottom.

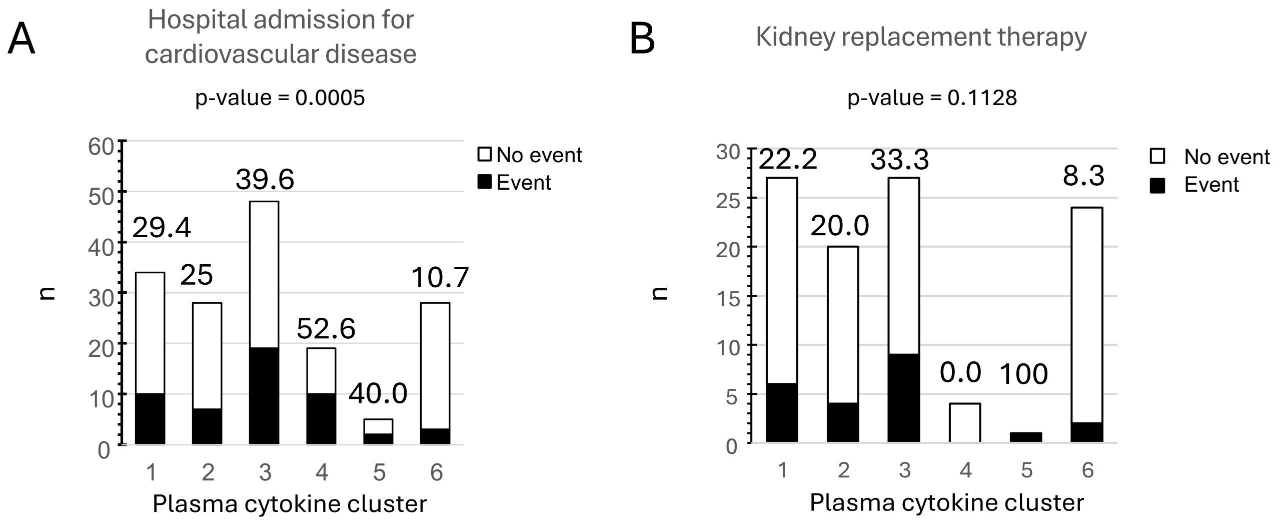

Fig. S8. Plasma cytokine clusters and short term (36 months) CVD progression and long-term (10 years) risk of KRT. **A)** Hospital admission for cardiovascular disease within 36 months. **B)** Kidney replacement therapy (KRT: dialysis or kidney transplantation) at 10 years, with deaths excluded from the calculation. The number on top of each column represents mortality expressed as a percentage of patients with known status. Statistical significance among the clusters was calculated using Fisher's exact test.
