# Supplementary material for: Systemic inflammatory profiles are associated with long-term kidney failure and patient mortality in chronic kidney disease
Source: Clin Kidney J. 2025 Dec 9;19(4):sfaf352. doi: 10.1093/ckj/sfaf352 (PMC13053082; doi:10.1093/ckj/sfaf352)
Supplement: sfaf352_Supplemental_Files [file sfaf352_supplemental_files.zip › Supplementary results O4.docx]

**Supplementary results**

**Baseline cytokine levels in each cytokine cluster**

Baseline cytokine levels were compared among clusters (**Figure S4**). Significant differences in baseline plasma levels of IL-8, IL-22, TNF-α, and GDF-15, which were used for cluster analysis, were observed among the clusters.

Among the cytokines not used for cluster analysis, plasma levels of IL-5, IL-6, IL-10, IL-17, IL-18, IL-23, CCL5, CCL17, CCL20, and CXCL9, as well as urinary levels of IL-22, CCL17, and GDF-15, showed significant differences across clusters.

In Cluster 6, which contained most healthy controls, the levels of markers consistently showed lower levels across the six clusters, except for plasma IL-5 levels and plasma and urinary CCL17 levels.

In Cluster 4, where plasma levels of IL-8, IL-22, TNF-α, and GDF-15 were all elevated, plasma levels of IL-6, IL-10, IL-18, CCL5, CCL17, CCL20, CXCL9, and urinary IL-22 levels were also significantly higher compared to Cluster 6.

In Cluster 3, characterized by low IL-8 levels, the levels of IL-18, IL-23, CCL17, CCL20, CXCL9, and urinary IL-22 were notably elevated, along with plasma levels of GDF-15, TNF-α, and IL-22.

In Cluster 1, only IL-8 levels were consistently elevated in all samples. However, in baseline samples, plasma levels of IL-22, TNF-α, GDF-15, IL-17, CCL20, and CXCL9 were also significantly higher compared to Cluster 6.

In Cluster 3, plasma levels of IL-6, IL-8, IL-10, and GDF-15 were significantly lower compared to Cluster 4.

In Cluster 2, only plasma IL-22 and TNF-α were higher than in Cluster 6.
